# High Prevalence of Antibiotic Resistance in Traditionally Fermented Foods as a Critical Risk Factor for Host Gut Antibiotic Resistome

**DOI:** 10.3390/microorganisms12071433

**Published:** 2024-07-15

**Authors:** Yutong Li, Siying Fu, Matthias S. Klein, Hua Wang

**Affiliations:** Department of Food Science and Technology, The Ohio State University, 2015 Fyffe Court, Columbus, OH 43210, USAmatthias.klein@mcgill.ca (M.S.K.)

**Keywords:** antibiotic resistance, artisan cheese, gut microbiota, intervention, kimchi, opportunistic pathogens, resistome, traditionally fermented foods

## Abstract

This study aimed to assess the suitability of fermented food interventions to replenish damaged gut microbiota. Metagenomic assessment of published sequencing data found that fermented food interventions led to a significant increase in the gut antibiotic resistome in healthy human subjects. Antibiotic resistome and viable antibiotic-resistant (AR) bacteria were further highly prevalent in retail kimchi and artisan cheeses by metagenomic and culture analyses. Representative AR pathogens of importance in nosocomial infections, such as *Klebsiella pneumoniae*, *Serratia marcescens*, and vancomycin-resistant *Enterococcus* (VRE), as well as commensals and lactic acid bacteria, were characterized; some exhibited an extremely high minimum inhibitory concentration (MIC) against antibiotics of clinical significance. Exposing fermented food microbiota to representative antibiotics further led to a boost of the corresponding antibiotic and multidrug-resistance gene pools, as well as disturbed microbiota, including the rise of previously undetectable pathogens. These results revealed an underestimated public health risk associated with fermented food intervention at the current stage, particularly for susceptible populations with compromised gut integrity and immune functions seeking gut microbiota rescue. The findings call for productive intervention of foodborne AR via technology innovation and strategic movements to mitigate unnecessary, massive damages to the host gut microbiota due to orally administered or biliary excreted antibiotics.

## 1. Introduction

The rapid rise of antibiotic resistance (AR) negates effective treatment of bacterial infections, shaking the foundation of modern medicine. AR bacteria in the host gastrointestinal tract further indirectly contribute to gut microbiota dysbiosis resulting from even short-term antibiotic treatment [1]. Gut microbiota destruction has been recognized as a critical, shared risk factor for a growing list of noncommunicable “modern” diseases, ranging from the malfunction of the host immune system, type-II diabetes, brain/neurological disorders, and *Clostridium difficile* infections to certain cardiovascular diseases and cancers [2,3,4,5,6,7,8,9,10]. Both AR and noncommunicable diseases are among the top global public health threats in the 21st century [11].

For decades, the broad applications of antibiotics have been blamed for the rapid surge of AR and, more recently, for disrupted host gut microbiota and associated “modern” diseases. However, despite limiting the use of antibiotics, which has been the primary control strategy worldwide, even essential antibiotic applications for infection prevention and treatment still cause irreversible damage to the host gut microbiota [12,13]. AR is a complicated issue with multiple risk factors [14,15]. Particularly, gut-impacting antibiotics, i.e., the mainstream oral administration of antibiotics that go through the intestines and using drugs (even by injection) that have primary biliary instead of renal excretion rather than the application of antibiotics, have been the key and shared driver for the rapid surge of the antibiotic resistance gene (ARG) pool, massively disrupted gut microbiota, and the rise of pathogens in gut microbiota [1,16,17,18]. This paradigm-changing discovery is further supported by clinical observations on the trends of AR worldwide [1,19]. The finding is also applicable to non-antibiotic drugs with an impact on the gut microbiota [20]. According to the CDC, over 210 million outpatient oral antibiotic prescriptions are given in the US annually [21]. Assuming even distribution, this is equivalent to over 60% of the U.S. population alone being affected every year, contributing to the global rising trends of the aforementioned diseases.

Given its impact on host health, various approaches to replenishing damaged gut microbiota have been attempted. Fecal microbiota transplant (FMT) is getting approval for treating *C. difficile* infections [22,23], but the procedure was associated with acquired infections, including death by ESBL *E. coli* [24]. It further led to a surge in resistome in both human and gnotobiotic pig recipients post-FMT procedures [25]. 

Fermented foods have emerged in recent years as a popular alternative to repair disrupted gut microbiota [26,27,28]. Wastyk et al. [28] reported increased diversity of the gut microbiota of the human recipients by consuming diets high in fermented foods but not by diets high in plant fibers. Fermented vegetables, followed by kombucha and fermented dairy products (yogurt and kefir), were among the most consumed categories of fermented foods by this group of subjects (Supplementary Materials Table S1).

However, fermented foods rich in viable microbes are susceptible to AR. Before 2007, fermented dairy foods had been the most significant foodborne avenue for transmitting AR to consumers [29]. A gram of retail cheese contained up to 10^8^ copies of AR genes [30]. Various bacteria isolated from fermented foods with mobile AR genes were able to cause acquired resistance in human commensals and pathogens via horizontal gene transfer mechanisms [29,31,32]. Although successful mitigation of the AR gene pool was quickly achieved in mainstream fermented dairy products by 2011, primarily by the removal of problematic fermentation starter cultures and probiotic strains from the product lines [14,33], other traditionally fermented products may still be prone to AR [34,35,36,37,38,39,40,41,42,43,44,45,46,47,48,49,50]. Unlike mainstream modern dairy fermentation, which uses pasteurized milk and commercial starter cultures carefully screened by major culture companies, traditionally fermented vegetables and artisan cheeses, for instance, still rely on microbiota associated with raw materials, environment, or “mother” cultures other than carefully screened commercial starters [34,35,39,42,43,44,46,48,50,51].

The fermented food and beverage market is expected to grow by 5.6% in the next 10 years and will exceed USD 989.2 billion by 2032 amid escalating demand for healthy and nutritious foods [52]. The COVID-19 pandemic has further facilitated the explosive growth of sales of fermented products such as sauerkraut and kimchi [53]. Given the booming market and increased consumption, it becomes crucial to know the impact of increased consumption of such fermented foods on host health. We hypothesized that traditionally fermented food products are rich in AR bacteria, and increased consumption of such products could lead to an increase in AR in the host gut microbiome. If true, this would represent a significant food safety and public health risk, especially for susceptible populations with compromised digestive tracts and immune systems. Therefore, the objectives of this study were to investigate the incidences of antibiotic resistome in representative retail fermented foods and to assess the impact of fermented food intervention on human gut resistome.

## 2. Methods and Materials

### 2.1. Overall Experimental Design

**Figure 1:**
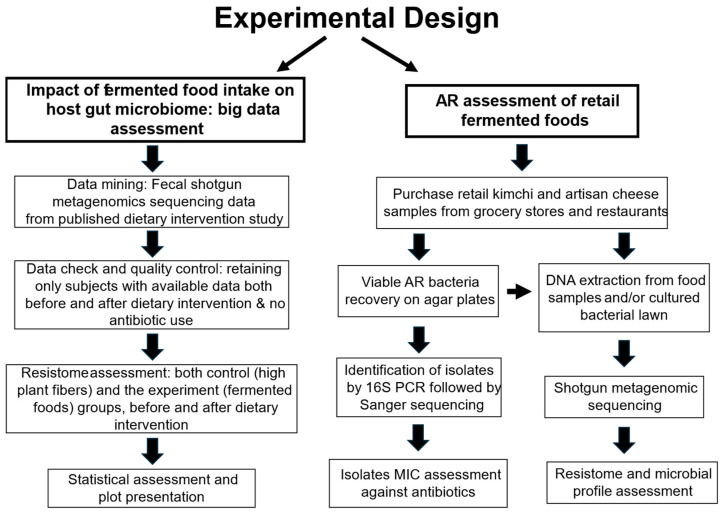
Experimental approach flowchart.

Figure 1 illustrates the overall experimental approaches to address the objectives, with detailed methods illustrated in the following sections.

### 2.2. Host Gut Resistome Assessment

In the original study by Wastyk et al. [28], a total of 39 healthy adult participants were recruited, with 18 randomly assigned to the dietary intervention group of fermented foods and the remaining 21 assigned to the plant fiber intervention group. Three participants in the fiber intervention group dropped out of the study; thus, both groups had 18 participants who finished the whole study (25 female, 11 male, with an average age of 52 ± 11 years) and without antibiotic treatments. Fecal shotgun metagenomics data of these participants were collected at four checkpoints, i.e., Week −2, Week 0, Week 8, and Week 10, throughout the study and deposited at the NCBI BioProject database (ID number: PRJNA743361). After initial screening for data availability, 3 participants in the fiber group and 1 participant in the fermented food group were dropped from our study due to missing Week 10 endpoint shotgun sequencing data in the database provided by the original research team. The raw sequencing data were retrieved from the database in Sequence Read Archive (.sra) format, converted to .fastq using the NCBI SRA Toolkit (version 3.0.0), and further processed on a high-performance supercomputer at the Ohio Supercomputer Center.

For quality control, the FASTP tool (version 0.22.0) was used (parameters: -q 20 -u 20 -n 2 -l 80) to clean and trim the raw sequences before further annotation and analysis, as described by Chen et al. [54]. The cleaned sequences were then processed using the ARGs-OAP pipeline (Online Analysis Pipeline for Antibiotic Resistance Genes, version 2.5) [55]. The resistome analysis criteria were set as a hit length of 50%, an e-value of 1 × 10^−07,^ and an identity of 80%, as recommended by the pipeline. The generated data were further analyzed and plotted using Tidyverse packages, including ggplot2 (version 3.3.6) and dplyr (version 1.0.10), ggstatsplot package (version 0.10.0), and car package (Companion to Applied Regression, version 3.1-1) on R version 4.2.1 [56,57,58,59,60].

### 2.3. Data Transformation, Assessment, and Statistical Analysis

The sums of the total ARG were calculated by adding up the relative abundances of all types of ARG annotated by ARGs-OAP (version 2.5). The percentage of total resistome change for each subject was calculated by the equation: (∑resistome reads after dietary intervention- ∑resistome reads before dietary intervention)/∑resistome reads before dietary intervention × 100%. The resistome data from Week −2 and Week 0 were averaged as the baseline to make sure all subjects had baseline data before dietary intervention and to reduce natural variation in gut microbiota. The normality and variance of data sets were verified using the Shapiro–Wilk normality test and Levene’s test. Violin-boxplots with Student’s *t*-test *p*-values (α < 0.05) were generated using the ggstatsplot package (version 0.10.0) [57]. Other plots were constructed using the ggplot2 package (version 3.3.6) [58]. Summary statistics such as mean, median, and standard deviation and the significance of dietary intervention were calculated using the dplyr package (version 1.0.10) [60].

### 2.4. Source of Fermented Food Samples

Representative kimchi samples of different brands were purchased from 7 retail stores, including independent operations and national chains, 1 chain restaurant, and 3 local Japanese and Korean restaurants in Columbus, OH, USA, from 2021–2023. The artisan cheese products were purchased from a national grocery chain store in Columbus, OH, with 4 purchased in 2021 and 4 in 2022. Sample designation and brief description were illustrated in Supplementary Materials Table S2.

### 2.5. Recovery and Assessment of Viable Bacteria from Fermented Food Samples

Five grams of each food sample were stomached in 45 mL of 0.1% peptone water using a Seward stomacher 80 (Seward, Worthing, UK). The juice was assessed for total bacteria and AR bacteria using Brain Heart Infusion (BHI), Luria–Bertani (LB), or De Man, Rogosa, and Sharpe (MRS) agar plates with cycloheximide or nystatin as mold inhibitors, with or without the corresponding antibiotic (16 or 32 µg/mL ampicillin; 32 or 64 µg/mL tetracycline; 2 µg/mL erythromycin for BHI or MRS). A control sample with only peptone water was processed under the exact same conditions and plated to make sure no contamination was introduced during the sample processing. The plates were incubated at 30 °C, aerobically or anaerobically. Single colonies recovered from antibiotic-containing agar plates were picked based on representation in morphology and identified by Sanger sequencing of 16S rRNA gene PCR amplicons [29]. The MIC of recovered AR isolates against four commonly used antibiotics was determined by a microdilution procedure [61] but using the corresponding recovery media instead. The MIC of some representative AR isolates against a broader spectrum of antibiotics was further determined using commercial antimicrobial susceptibility kits Sensititre^®^ (Thermo Scientific, Waltham, MA, USA) GPN3F for Gram-positive isolates and GN3F for Gram-negative isolates, following the manufacturer’s instructions, with MRS or BHI broth replacing the Sensititre Mueller Hinton broth for better growth of each microbe. Clinical and Laboratory Standards Institute (CLSI) [62] control strains *Escherichia coli* ATCC 25922 and *Enterococcus faecalis* 29212 (ATCC, Manassas, VA, USA) were used as the MIC control standards.

### 2.6. Antibiotic Resistome of Representative Fermented Food Items

Twenty-five grams of food samples were mixed with 25 mL of 0.1% peptone water and stomached as above. The large particles of food debris were removed with a sterile sieve. The liquid phase was centrifuged at 2500 relative centrifugal force by Multifuge X1R (Thermo Scientific, Waltham, MA, USA) for 15 min. The pellet was washed once with 0.1% peptone water and subjected to total DNA extraction with a QIAamp PowerFecal Pro DNA Kit (Qiagen, Hilden, Germany) following the manufacturer’s instructions. Alternatively, the culture-recovered microbiota was scraped from agar plates with 3 mL of 0.1% peptone water and subjected to DNA extraction using the aforementioned QIAamp kit (Qiagen, Hilden, Germany).

The DNA samples were subjected to shotgun metagenomic sequencing (2 × 155 bp) with an average depth of 16.7 to 20 million paired-end reads for each sample, using an Illumina NextSeq2000 Sequencing System (Illumina, San Diego, CA, USA). The raw sequences were cleaned (parameters: -q 20 -u 40 -n 2 -l 80) and analyzed with the same metagenomic tools and parameters as described above to obtain a relative abundance of AR genes normalized to the number of 16S rRNA genes. The sequencing data were further assessed for microbiota profiles through the Kraken2 pipeline (version 2.1.2) with a standard database and minimum base quality of 20, as described previously [63]. The food microbiota profile of Kimchi #7 was also assessed by 16S rRNA sequencing by an Illumina MiSeq system (Illumina, San Diego, CA, USA).

All local metagenomic data analyses were conducted on a high-performance supercomputer at the Ohio Supercomputer Center.

## 3. Results

### 3.1. Fermented Food Intervention Led to the Rise of the Host Fecal Microbiota Resistome

**Figure 2:**
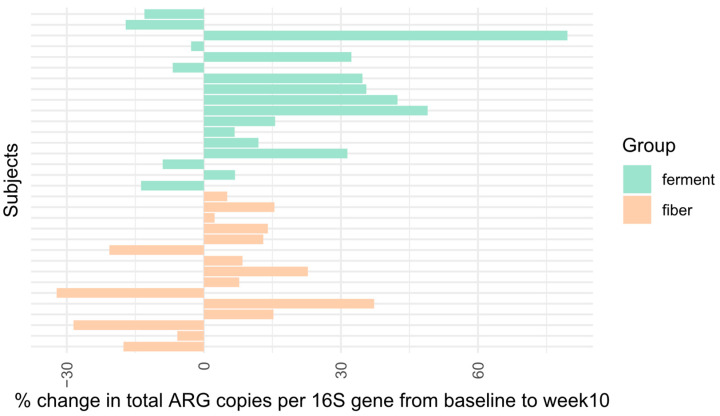
Impact of a 10week food intervention on the change in antibiotic resistome of the fecal microbiota of human subjects in percentage compared to the baseline. Green: foods rich in fermented products; orange: foods rich in plant fibers.

**Figure 3:**
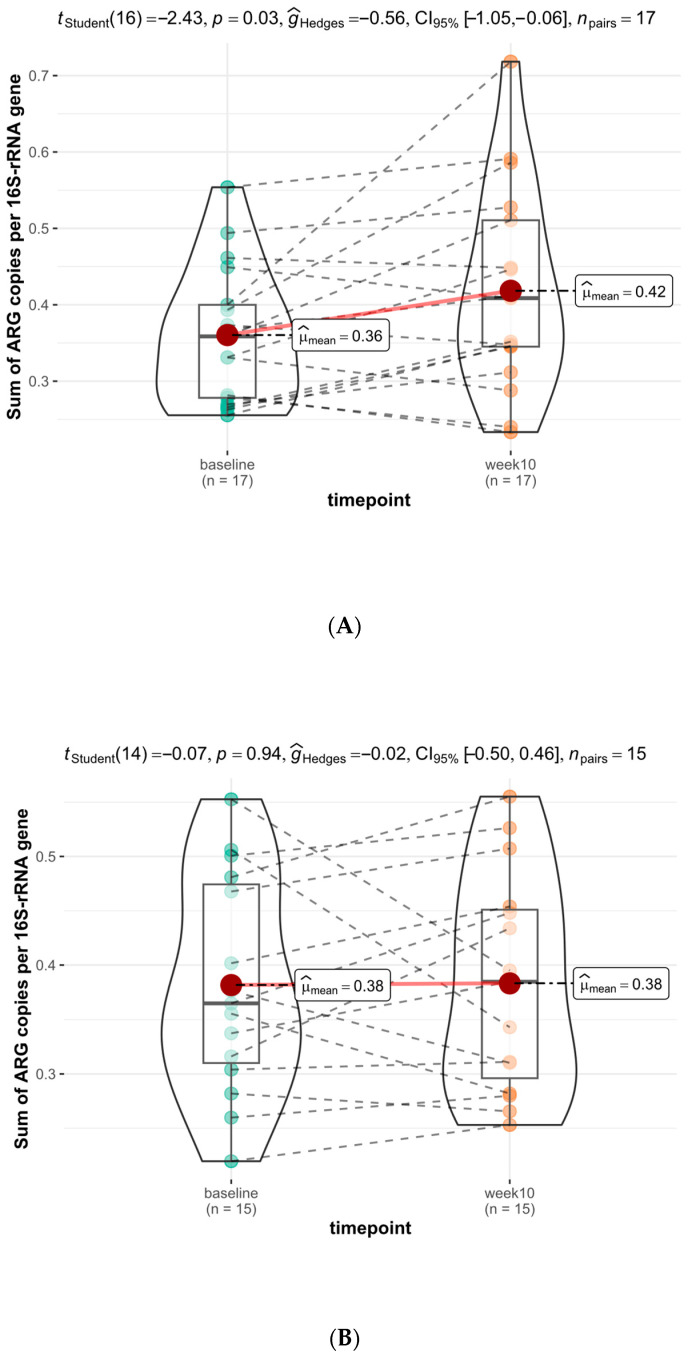
Violin-boxplots with Student’s *t*-test *p*-values illustrate the impact of a 10-week food intervention on the change in antibiotic resistome in the fecal microbiota of human subjects compared to the baseline by diets rich in (**A**) fermented foods and (**B**) plant fibers.

Ten-week dietary interventions with diets high in fermented foods or plant fibers led to 11 increases and 6 decreases, as well as 10 increases and 5 decreases in gut antibiotic resistome in human subjects, respectively, but the impact of the two types of diets was distinctive (Figure 2 and Figure 3). Fermented food intervention led to significant changes in the fecal antibiotic resistome of subjects with a *p*-value of 0.03, and the mean of resistome increased from 0.36 to 0.42 copies/16S rRNA gene (Figure 3A). Meanwhile, intervention by high-plant-fiber diets caused an insignificant change in the host gut resistome, with a p-value of 0.94. The mean of resistome remained at 0.38 copies/16S rRNA gene in subjects before and after dietary intervention (Figure 3B). The summarized resistome data used are illustrated in Supplementary Materials Table S3. For details of the resistome data, see Supplementary Materials Tables S9 and S10.

The data suggested that the significantly increased gut antibiotic resistome in subjects with fermented food interventions was likely due to the rich, viable foodborne AR bacteria in fermented foods. The food macromolecules (ingredients), as consumed by subjects in the control group with dietary intervention from high-fiber foods, did not impact gut antibiotic resistome.

### 3.2. Antibiotic Resistome in Traditionally Fermented Foods

This pilot screening of retail fermented foods included products from diversified brands distributed nationwide through national and regional grocery chains and independent retailers.

#### 3.2.1. Antibiotic Ressitome Highly Prevalent but Diversified in Retail Kimchi Samples

**Figure 4:**
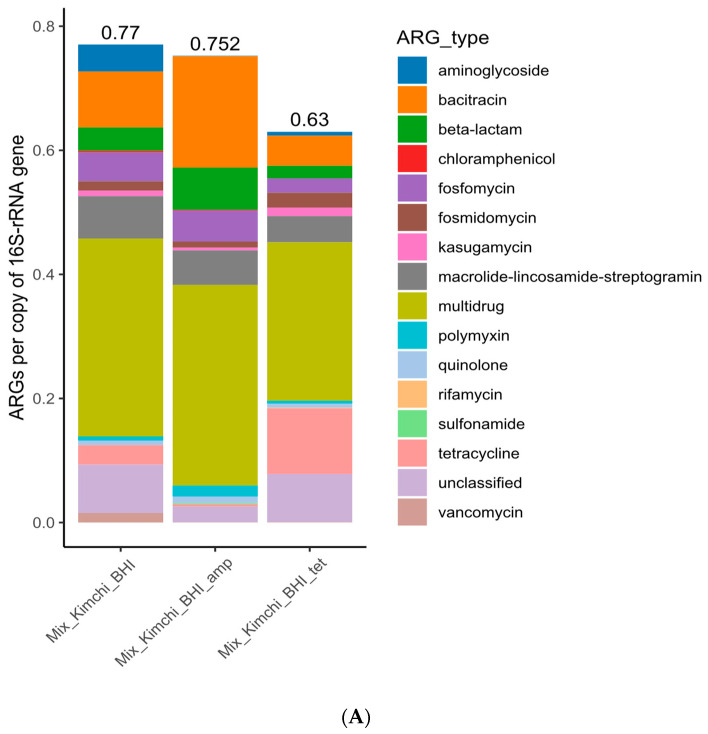

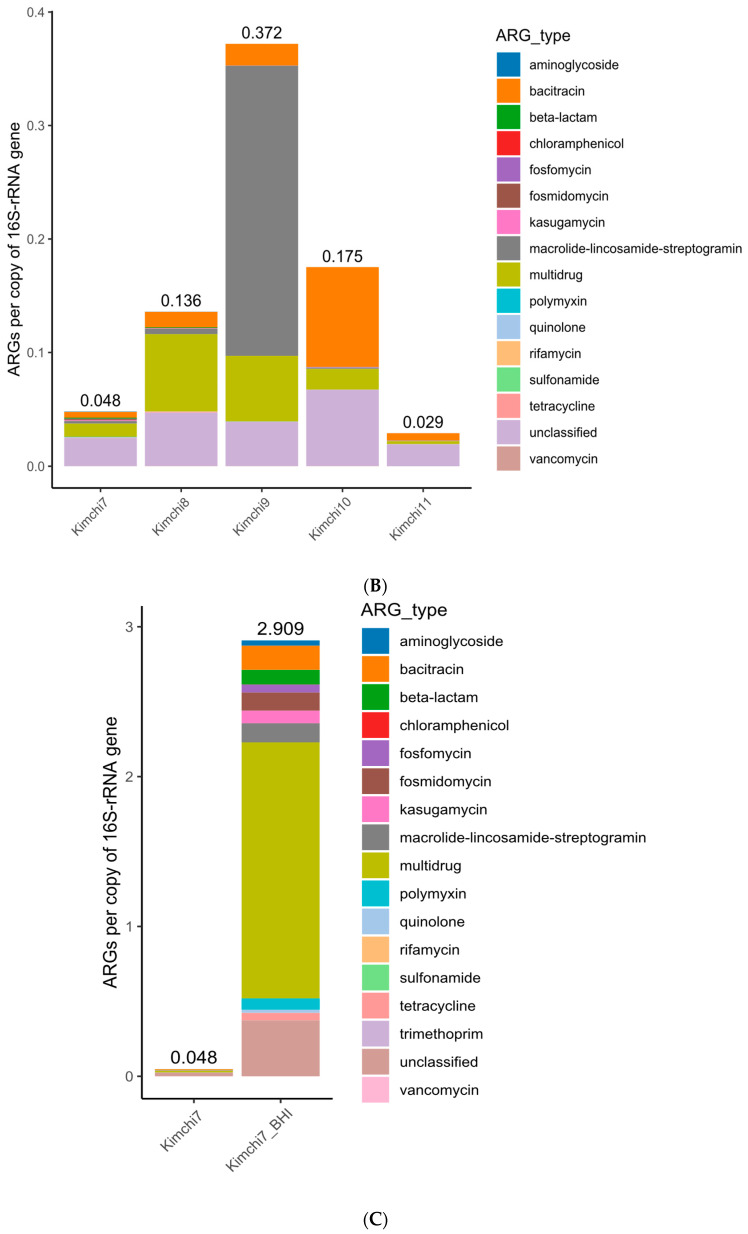
Antibiotic resistome of (**A**) pooled food microbiota of 4 kimchi samples (K1–K4) purchased in 2021, recovered from BHI agar plates; (**B**) direct microbiota (DNAs extracted) of kimchi samples purchased in 2022; (**C**) direct kimchi microbiota (left bar) and BHI agar plate-recovered microbiota (right bar) of kimchi sample 7.

Multidrug resistance, bacitracin, and macrolide–lincosamide–streptogramin (MLS) genes were most abundant in pooled retail kimchi samples (K1–K4) recovered from BHI plates (Figure 4A and Table 1), and they were also among the most abundant AR genes of all the five individual kimchi microbiota (K7–K11) assessed directly, despite the fact that the exact abundance of the top AR genes varied among these samples (Table 1 and Figure 4B).

Likewise, even though AR isolates were identified in 9 out of 10 kimchi samples illustrated (K5, K7–K14, Table 2), the overall abundance of the resistome of the assessed samples also varied, ranging from 0.372 (K9) to 0.029 (K11) copies of AR genes/16S rRNA (Figure 4B).

#### 3.2.2. Sample Analysis Approach Affected the Outcome Details of Kimchi Microbiome Assessments

Figure 4C further compares the resistome outcomes of kimchi sample K7 using total DNA extracted directly from the kimchi microbiota (0.048 copies of AR genes/16S) and the total DNA of the kimchi microbiota recovered from the BHI agar plate (2.909 copies of AR genes/16S). The results indicated that with specific medium and incubation conditions, even without antibiotic selective pressure, certain bacteria of the kimchi microbiota might have been selectively enriched, leading to the drastic difference in detected antibiotic resistome. Likewise, the top genera of pooled kimchi microbiota recovered from BHI plates (Supplementary Materials Table S4) were affected by cultivation conditions too, with and without antibiotics, as illustrated by the resistome in Figure 4C.

#### 3.2.3. Antibiotic Resistome Prevalent Even in Successfully Fermented Kimchi Samples

Moreover, Table 3 illustrates that the top genera of each individual kimchi microbiota (K7 to K11) assessed using total DNA extracted directly were dominated by lactic acid bacteria well-recognized for driving kimchi fermentation. The data further demonstrated that antibiotic resistome and AR bacteria were still prevalent in these successfully fermented kimchi samples.

#### 3.2.4. Antibiotic Resistome Highly Prevalent in Artisan Cheeses

**Figure 5:**
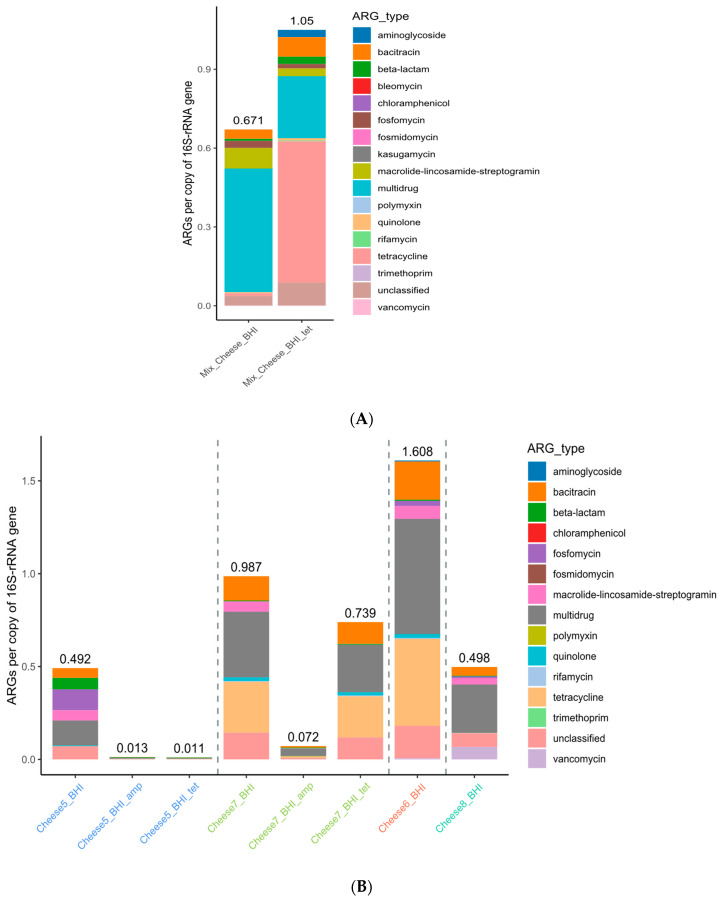
Antibiotic resistome of (**A**) pooled food microbiota recovered from BHI agar plates of 4 artisan cheeses (C1–C4) purchased in 2021; (**B**) BHI plate-recovered microbiota of artisan cheeses purchased in 2022.

The resistome results of pooled artisan cheese samples C1 to C4 (Figure 5A) and individual cheese samples C5 to C8 (Figure 5B), all using microbiota recovered from BHI agar plates, further illustrated the prevalence of various AR genes in these products. Multidrug, MLS, and bacitracin were among the top AR genes of the pooled samples C1–C4, while multidrug, bacitracin, tetracycline, and MLS were most abundant among individual samples C5 to C8 (Table 4). It is worth noting that, similar to the previously discussed kimchi samples, the resistome abundance of cheese microbiota recovered from BHI plates was likely also affected by the medium and culture conditions, which deviated from those of the original samples.

Supplementary Materials Table S6 further illustrates that *Staphylococcus* (including *Mammaliicoccus*, also known as *Staphylococcus*) was the top dominant genus of individual artisan cheese microbiota recovered from BHI agar plates.

#### 3.2.5. Viable AR Bacteria Are Highly Prevalent in Traditionally Fermented Foods

Various AR bacteria were recovered from traditionally fermented products. Under very limited cultivation conditions applied in this study, AR bacteria were identified from 9 out of 10 kimchi and 4 out of 4 cheese samples assessed by culture recovery (Table 2 and Table 5).

Identified AR isolates from kimchi products ranged from important human pathogens to organisms still considered commensals, as well as lactic acid bacteria driving food fermentation. For instance, AR opportunistic pathogens such as *Klebsiella pneumoniae* and *Serratia marcescens* are recognized agents for infections [66,67]. *Rahnella aquatilis*, as a human pathogen, can cause bacteremia, sepsis, urinary tract infections, etc. As illustrated in Table 2 and Supplementary Materials Table S7, kimchi isolates *Klebsiella pneumoniae* K7-6 and *Rahnella aquatilis* K11-3 are resistant to multiple antibiotics. Particularly, *Serratia marcescens* K13-1 is highly resistant to almost all key antibiotics (21 out of 22), including the 4th generation of cephalosporin antibiotic as well as carbapenem antibiotics, with MICs exceeding the highest detection limit of 12 antibiotics by the Sensititre assessment (Supplementary Materials Table S7). In addition, isolates of several species of lactic acid bacteria, such as *Latilactobacillus* and *Leuconostoc*, commonly involved in driving kimchi fermentation, were among those highly resistant to tetracycline, erythromycin, ampicillin, and vancomycin, and some isolates exhibited multidrug resistance (Table 2). Even within the same genus and species of lactic acid bacteria, certain isolates may be resistant to more antibiotics than others. For instance, *Lb. sakei* K9-3 exhibited resistance to 17 out of 18 antibiotics examined, except tetracycline, and about half at the highest MIC levels using the Sensititre panel, while *Lb. sakei* K5-1 was resistant to 13 out of 18 antibiotics examined, including tetracycline (Supplementary Materials Table S8).

Identified AR isolates from cheese products were mostly *Staphylococcus* spp. resistant to tetracycline or erythromycin, with one isolate also resistant to vancomycin. An isolate of *Enterococcus faecalis* was multidrug-resistant, including highly resistant to vancomycin (Supplementary Materials Table S8).

#### 3.2.6. Fermented Food-Originated Microbiota Can Be a Source of Many Drug-Resistant Pathogens When Exposed to Antibiotics

**Figure 6:**
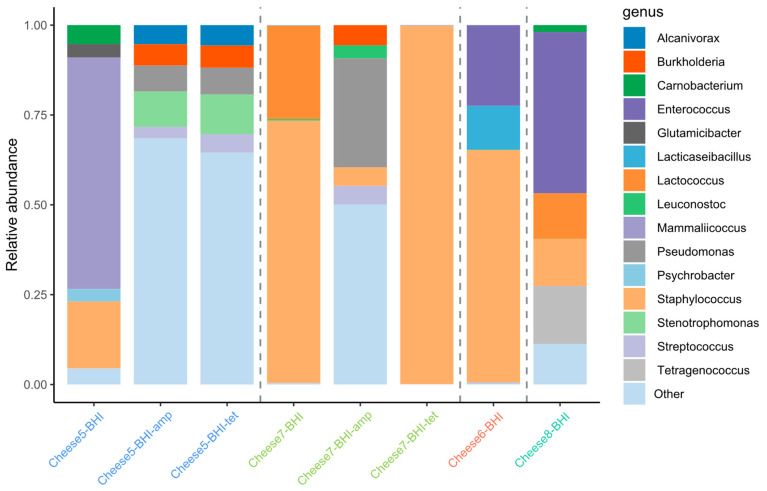
Detected cheese microbiota recovered from BHI agar plates with and without antibiotics.

As illustrated in Figure 6 and Supplementary Materials Table S6, cheese microbiota harvested from BHI plates without antibiotics were dominated by *Mammaliicoccus* spp. (formerly *Staphylococcus*), *Staphylococcus*, *Camobacterium*, *Psychrobacter,* and *Glutamicibacter* (belonging to *Micrococcaceae*) for cheese sample C5. However, in the presence of Tet or Amp, the dominant bacteria switched to *Stenotrophomonas*, *Pseudomonas*, *Burkholderia*, *Alcanivorax,* and *Streptococcus*. Likewise, while the dominant cultures for cheese C7 recovered on BHI were *Staphylococcus* and *Lactococcus*, the top-ranked bacteria of cheese-originated microbiota shifted to *Pseudomonas*, *Burkholderia*, *Staphylococcus*, *Streptococcus*, and *Leuconostoc* on BHI-Amp and *Staphylococcus* on BHI-Tet. In the case of C7 and pooled sample C1–C4, the cheese starter culture *Lactococcus* was essentially eliminated when the cheese microbiota was exposed to either of the two antibiotics.

Although antibiotic-containing agar plates were used to screen for AR bacteria, the above results also indicated how the fermented food-originated microbiota might impact the gut microbiota when the hosts received antibiotic treatment. *Stenotrophomonas*, *Pseudomonas*, *Burkholderia*, and *Staphylococcus* are recognized opportunistic pathogens associated with antibiotic-resistant hospital-acquired infections, and most *Streptococcus* species are pathogens.

Microbial profiling by metagenomics of dominant cheese bacteria recovered on BHI plates included *Staphylococcus*, *Enterococcus,* and *Lactocaseibacillus* (formerly *Lactobacillus*) for cheese sample C6, as well as *Enterococcus*, *Tetragenococcus*, *Staphylococcus*, *Lactococcus,* and *Camobacterium* for sample C8, respectively (Figure 6 and Supplementary Materials Table S6). In agreement, some of the mentioned antibiotic-resistant bacteria, such as multidrug-resistant *Enterococcus* and *Staphylococcus,* were also confirmed in these samples (Table 5).

## 4. Discussion

Although unexpected for many, the susceptibility of fermented foods to AR has been well-recognized, at least in the food microbiology community. Since the first systematic demonstration of the problem in the early 2000s with a broad spectrum of AR isolates, including starter cultures, opportunistic pathogens, and commensals of mainstream fermented dairy products being identified and characterized [29], AR bacteria have further been isolated from various fermented foods worldwide [68,69,70]. For instance, *Pantoea agglomerans* (formerly *Enterobacter agglomerans* or *Erwinia herbicola*), an opportunistic pathogen causative of a wide range of opportunistic infections, especially in immunocompromised patients, was isolated from kimchi in South Korea. The genome of *P. agglomerans* isolate KM1 contained 13 genes conferring resistance to clinically important antibiotics, and the strain exhibited immunostimulatory properties in vitro, including the production of pro-inflammatory and anti-inflammatory cytokines in stimulated cells [71].

This study, however, using a combination of approaches, illustrated the high prevalence and abundance of antibiotic resistome in popular traditionally fermented foods. Although the retail products in this report were purchased in Columbus, Ohio, the products were mostly made in the US and distributed nationwide through independent stores and grocery chains. Therefore, they serve as a good indication of the prevalence of AR in similar products nationwide. In fact, the prevalence and abundance of AR bacteria were also confirmed in representative retail kimchi of the same brands used by the subjects in the Wastyk study [28], purchased from Northern California. Our team has further conducted traditional kimchi fermentation carefully in a microbial-controlled setting and has concluded that AR is inevitable in the final products due to the AR bacteria associated with the raw vegetable materials. This is consistent with the principle of fermentation, which states that the natural microbiota from raw materials drives microbial succession in natural fermentation.

The original human study on dietary intervention by Wastyk et al. [28] had a total of 36 healthy adult subjects complete the study; half received fermented foods, and the other half received dietary fiber intervention. Among those, the fecal microbiome outcomes of 32 subjects with the required data points, including 17 subjects with a fermented food intervention and 15 subjects with a diet high in plant fiber for comparison, were assessed and reported for the impact of the dietary intervention on the host gut resistome in this study.

The sample size was still small, given the diversity of human subjects and activities. Nevertheless, the illustration of antibiotic resistome in the kimchi and artisan cheese microbiomes, along with the high prevalence of confirmed AR bacteria in kimchi and artisan cheeses assessed in this pilot study, support the conclusion that consuming these traditionally fermented products would result in the rise of gut antibiotic resistome in consumers. Since only limited conditions were applied in cultivating AR bacteria in this pilot study, the recovered isolates, in fact, only represent a small percentage of the AR microbiota of the fermented foods.

It is further worth noting that even in subjects who consumed only yogurt and kombucha (Subject 8020) or yogurt, kombucha, and kefir (Subject 8014) (Table S3), the gut resistome of the subjects still increased by 11% and 17%, respectively, after the 10-week intervention. The data suggest that consuming these products rich in conventional probiotics, at least, did not reduce the gut antibiotic resistome in the subjects.

The identification of AR bacteria from traditionally fermented products, ranging from human and plant pathogens to lactic acid bacteria driving food fermentation, especially those that exhibited multidrug resistance and had extremely high MICs against antibiotics of clinical significance, is particularly concerning. The multidrug-resistant isolate of *Enterococcus faecalis* from artisan cheese, also highly resistant to vancomycin, could be classified as vancomycin-resistant *Enterococcus* (VRE) [72]. Multidrug-resistant *Klebsiella pneumoniae*, *Serratia marcescens*, vancomycin-resistant *Enterococcus* (VRE), as well as *Stenotrophomonas*, *Pseudomonas*, *Burkholderia*, *Staphylococcus,* and *Streptococcus,* are also known for causing hospital-acquired infections, including some of the most troublesome cases in healthcare facilities. *Rahnella aquatilis* is a rare opportunistic pathogen associated with disease in immunosuppressed individuals. Data from this study thus also provide an alternative interpretation of the route of dissemination and potential origins of infections in patients. For instance, *Serratia marcescens* K13-1 is extremely resistant to most antibiotics assessed by the Sensititre panel for Gram-negative bacteria, including the carbapenem antibiotics Meropenem and Ertapenem that are usually reserved for treating multidrug-resistant bacterial infections (Supplementary Materials Table S7). *Lactobacillus sakei* K9-4 is further resistant to 17 out of 18 antibiotics (except tetracycline), including the 3rd generation of cephalosporin antibiotic ceftriaxone, assessed by the Sensititre panel for Gram-positive bacteria (Supplementary Materials Table S8). Meanwhile, within the *Lactobacillus* genus, *Lb sakei* K5-1 and *Lb. curvatus* K9-1 exhibited resistance to 13 or more antibiotics, including tetracycline, with diversified MIC levels. The data illustrated that various AR genes likely contributed to most of the observed AR profiles in these multidrug-resistant bacteria instead of innate resistance. Revealing the genetic elements responsible for the unusual AR profiles and their potential involvement in horizontal gene transfer will provide further insights into the food safety and public health risks associated with these AR bacteria.

The balanced gut microbiota, as well as effective mucosal, intestinal epithelial, and gut vascular barriers as part of an integral and functional gastrointestinal tract system, are essential to host health [73]. Physical damage to the intestinal barrier has various health consequences. Intestinal tract problems such as inflammatory bowel diseases, enteric infections, or overgrowth by pathogens such as EHEC and Shiga toxin-producing *E. coli*, *Shigella*, *Salmonella*, *Campylobacter*, *C. difficile*, enteric viruses, certain drug treatments such as the antibiotic clindamycin, etc., or even alcohol consumption can all cause acute or chronic intestinal barrier damages. Once the intestinal barriers collapse, intestinal microorganisms and their metabolites can enter the bloodstream, reach other organs, and cause further health damage. Once entering the bloodstream, even commensals and probiotics can be problematic. For instance, the most popular probiotic strains, *Bifidobacterium* and *L. rhamnosus* GG, have still been found to cause bacteremia and sepsis in high-risk populations, with strain confirmation by metagenomic sequencing [17,74,75,76,77,78,79,80,81,82]. While bacteremia and sepsis caused by commercial probiotic strains absence of AR genes are still controllable by mainstream antibiotics such as ampicillin, introducing the broad spectrum of AR bacteria highly resistant to clinically important antibiotics associated with traditionally fermented foods presents an underestimated and potentially serious public health risk, especially to targeted populations suffering from various gut symptoms associated with “leaky gut” as well as compromised immune functions. Bacteremia and sepsis risk, antibiotic therapy failures, and further gut microbiota destruction post-antibiotic treatment may significantly increase in the targeted susceptible consumer populations who intend to repair damaged gut microbiomes by enhancing fermented food consumption.

## 5. Conclusions and Perspectives

Data from this study, for the first time, comprehensively revealed several critical facts. First, various antibiotic-resistant genes and viable AR bacteria, including multidrug-resistant pathogens of significance to nosocomial infections, as well as AR lactic acid bacteria and other commensals, are highly prevalent in traditionally fermented foods. Second, fermented food microbiota can serve as a source of a broad and diversified spectrum of antibiotic-resistant pathogens of significance to nosocomial infections when exposed to antibiotics. Moreover, dietary intervention with fermented foods in the current state has led to a significant rise in gut antibiotic resistome in consumers. Thus, increased consumption of such traditionally or naturally fermented foods rich in AR bacteria, including pathogens, would represent a significant and underestimated food safety and public health risk, especially to patients suffering from “leaky” gut and malfunction of the immune system. Targeted mitigation of AR in such fermented foods by fermentation and processing technology innovation has thus become an urgent need.

With the recipients of over 210 million oral antibiotic prescriptions added to the susceptible population suffering from broad gut microbiota destruction annually in the US alone [21], the impacted patient population worldwide is astonishing. While various gut microbiota-replenishing approaches, including fermented food intervention, have been practiced desperately, they all have introduced additional public health risks to the patients. Results from this study thus further call for strategic efforts to tackle the key and shared driver of the antibiotic resistome surge and gut microbiota disruption in humans and animals by mitigating the applications of gut-impacting antibiotics (oral administration and biliary excretion) and offering alternatives to significantly minimize unnecessary damages to the gut microbiome for productive outcomes.

## Figures and Tables

**Table 1:** Most abundant AR genes in kimchi samples *.

Sample #	Rank				
	1	2	3	4	5
K1–K4 (BHI, pooled)	Multidrug 0.3189 *	Bacitracin 0.0901	Unclassified 0.0780	MLS ** 0.0684	Fosfomycin 0.0465
K1–K4 (BHI-Tet, pooled)	Multidrug 0.2556	Tetracycline 0.1059	Unclassified 0.0777	Bacitracin 0.0485	MLS 0.0422
K1–K4 (BHI-Amp, pooled)	Multidrug 0.3235	Bacitracin 0.1795	β-lactam 0.0680	MLS 0.0557	Fosfomycin 0.0498
K7 (BHI)	Multidrug 1.7085	Unclassified 0.3728	Bacitracin 0.1609	MLS 0.1278	Fosmidomycin 0.1216
K7	Unclassified 0.0248	Multidrug 0.0118	Bacitracin 0.0050	MLS 0.0023	Fosmidomycin 0.0012
K8	Multidrug 0.0681	Unclassified 0.0469	Bacitracin 0.0136	MLS 0.0047	Tetracycline 0.0006
K9	MLS 0.2557	Multidrug 0.0577	Unclassified 0.0390	Bacitracin 0.0191	Vancomycin 0.0001
K10	Bacitracin 0.0880	Unclassified 0.0669	Multidrug 0.0182	MLS 0.0009	Fosfomycin 0.0005
K11	Unclassified 0.0191	Bacitracin 0.0063	Multidrug 0.0026	Tetracycline 0.0002	MLS * 0.0002

* numerical readings of AR: in total ARG copies per 16S gene. ** MLS: macrolide–lincosamide–streptogramin.

**Table 2:** Summary of identified AR colonies from kimchi products purchased in 2022 and 2023 *.

Isolate	Identity	Food Source	Recovered From	Resistance with MIC * (µg/mL)
K5-1 ***	*Latilactobacillus sakei*	Kimchi 5	MRS-tet	Tet (>64) Van (>128)
K5-2	*Bacillus (licheniformis/paralicheniformis)*	Kimchi 5	BHI-ery	Amp (>64) Ery (>16)
K7-5	*Serratia quinivorans*	Kimchi 7	BHI/BHI-amp	Amp (>128) Van (>1024) **
K7-6 ***	*Klebsiella pneumoniae*	Kimchi 7	BHI/BHI-amp	Amp (>128) Ery (>100) ** Van (>1024) **
K7-8	*Enterobacter* sp.	Kimchi 7	BHI/BHI-amp	Amp (>128) Ery (>100) ** Van (>1024) **
K7-9	*Rahnella aquatilis*	Kimchi 7	BHI/BHI-amp	Amp (>128) Ery (>100) ** Van(>1024) **
K8-6	*Bacillus licheniformis*	Kimchi 8	BHI-ery	Amp (>32) Ery (>16)
K9-1 ***	*Latilactobacillus curvatus*	Kimchi 9	MRS-tet	Tet (>64) Van (>128)
K9-2	*Bacillus (licheniformis/paralicheniformis)*	Kimchi 9	BHI-ery	Amp (>16) Ery (>16)
K9-3 ***	*Latilactobacillus sakei*	Kimchi 9	MRS-ery	Ery (>16) Van (>128)
K10-1 ***	*Levilactobacillus brevis*	Kimchi 10	BHI-tet	Tet (>8) Van (>128)
K10-2	*Levilactobacillus brevis*	Kimchi 10	BHI-tet	Tet (>8) Van (>128)
K11-3 ***	*Rahnella aquatilis*	Kimchi 11	MRS-amp	Amp (>128) Van (>512) **
K12-1 ***	*Leuconostoc citreum*	Kimchi 12	BHI-tet	Tet (>64) Van (>128)
K12-6	*Latilactobacillus sakei*	Kimchi 12	BHI-tet	Tet (>32) Van (>128)
K13-1 ***	*Serratia marcescens*	Kimchi 13	BHI-tet	Amp (>128) Ery (>200) ** Tet (>128) Van (>1024) **
K13-2	*Oceanobacillis jeddahense*	Kimchi 13	BHI-tet	Tet (>32) Ery (>16)
K14-2 ***	*Levilactobacillus brevis*	Kimchi 14	MRS/MRS-tet	Tet (>16)

* Tested MIC for representative antibiotics, including Van: vancomycin; Amp: ampicillin; Ery: Erythromycin; Tet: Tetracycline. ** Likely due to the natural resistance of most Gram-negative bacteria against vancomycin and erythromycin [64,65]. *** MIC of this isolate against a broader spectrum of antibiotics was determined with the Sensititre^®^ kit available in Supplementary Materials Tables S7 and S8.

**Table 3:** Top five genera were classified as microbiota directly from five kimchi samples purchased in 2022.

Sample	Top Genera	Percentage
Kimchi7	*Leuconostoc*	94.87%
	Other	3.17%
	*Rahnella*	0.85%
	*Weissella*	0.48%
	*Levilactobacillus*	0.36%
	*Xanthomonas*	0.27%
Kimchi8	*Latilactobacillus*	55.98%
	*Leuconostoc*	28.90%
	*Lactiplantibacillus*	7.49%
	Other	5.46%
	*Lactococcus*	1.59%
	*Levilactobacillus*	0.58%
Kimchi9	*Latilactobacillus*	81.60%
	*Leuconostoc*	11.79%
	Other	3.45%
	*Weissella*	2.02%
	*Lactiplantibacillus*	0.59%
	*Levilactobacillus*	0.55%
Kimchi10	*Lactiplantibacillus*	54.60%
	*Leuconostoc*	32.89%
	*Levilactobacillus*	9.80%
	Other	1.86%
	*Weissella*	0.55%
	*Latilactobacillus*	0.30%
Kimchi11	*Weissella*	58.85%
	*Leuconostoc*	39.19%
	Other	1.41%
	*Latilactobacillus*	0.23%
	*Lactiplantibacillus*	0.19%
	*Stenotrophomonas*	0.13%

**Table 4:** Most abundant AR genes in BHI plates recovered the microbiota of artisan cheese samples.

Sample #	Rank				
	1	2	3	4	5
C1–C4 (BHI, pooled)	Multidrug 0.4704 *	MLS ** 0.0783	Bacitracin 0.0353	Fosfomycin 0.0265	Tetracycline 0.0105
C1–C4 (BHI-Tet, pooled)	Tetracycline 0.5377	Multidrug 0.2359	Unclassified 0.0876	Bacitracin 0.0744	MLS 0.0295
C1–C4 (BHI-Amp, pooled)	Multidrug 0.0308	Tetracycline 0.0093	MLS 0.0084	Bacitracin 0.0053	Unclassified 0.0033
C5 (BHI)	Multidrug 0.1343	Fosfomycin 0.1123	Unclassified 0.0709	β-lactam 0.0617	MLS 0.0555
C5 (BHI-Tet)	Multidrug 0.0033	β-lactam 0.0031	Tetracycline 0.0014	Bacitracin 0.0012	Unclassified 0.0012
C5 (BHI-Amp)	Multidrug 0.0040	β-lactam 0.0038	Unclassified 0.0015	Bacitracin 0.0014	MLS 0.0013
C6 (BHI)	Multidrug 0.6202	Tetracycline 0.4674	Bacitracin 0.2046	Unclassified 0.1750	MLS 0.0694
C7 (BHI)	Multidrug 0.3538	Tetracycline 0.2740	Unclassified 0.1442	Bacitracin 0.1308	MLS 0.0557
C7 (BHI-Tet)	Multidrug 0.2558	Tetracycline 0.2215	Unclassified 0.1183	Bacitracin 0.1176	Quinolone 0.0167
C7 (BHI-Amp)	Multidrug 0.0394	Bacitracin 0.0085	Tetracycline 0.0078	Unclassified 0.0075	β-lactam 0.0035
C8 (BHI)	Multidrug 0.2605	Unclassified 0.0737	Vancomycin 0.0671	Bacitracin 0.0472	MLS 0.0345

* numerical readings in total ARG copies per 16S gene. ** MLS: macrolide–lincosamide–streptogramin.

**Table 5:** Summary of identified AR colonies from artisan cheese products purchased in 2022 *.

Isolate	Identity	Food Source	Recovered From	Resistance with MIC * (µg/mL)
C5-3	*Staphylococcus equorum*	Cheese 5	BHI-ery	Ery (>16)
C5-9	*Mammaliicoccus vitulinus*	Cheese 5	BHI-ery	Ery (>8)
C6-2 **	*Staphylococcus xylosus*	Cheese 6	BHI-tet	Tet (>16)
C6-4	*Staphylococcus saprophyticus*	Cheese 6	BHI-tet	Tet (>32)
C6-10	*Staphylococcus xylosus*	Cheese 6	BHI-tet	Tet (>32) Van (>128)
C6-11 **	*Enterococcus faecalis*	Cheese 6	MRS-ery	Ery (>4) Van (>128)
C7-1	*Staphylococcus xylosus*	Cheese 7	BHI-tet	Tet (>8)
C7-5	*Staphylococcus saprophyticus*	Cheese 7	BHI-tet	Tet (>32)
C8-11	*Staphylococcus equorum*	Cheese 8	BHI-ery	Ery (>16)

* Tested MICs for representative antibiotics include Van: vancomycin; Amp: ampicillin; Ery: Erythromycin; Tet: Tetracycline. ** MIC of this isolate against a broader spectrum of antibiotics was determined with the Sensititre^®^ kit available in Supplementary Materials Tables S7 and S8.

## Data Availability

All metagenomic sequencing data were deposited in the NCBI BioProject database (ID number: PRJNA944373).

## Supplementary Materials

The following supporting information can be downloaded at: https://www.mdpi.com/article/10.3390/microorganisms12071433/s1. Supplemental Table S1. Categories of foods consumed by subjects for dietary intervention. Supplemental Table S2. Fermented food samples assessed in this study. Supplemental Table S3. Gut antibiotic resistome of subjects with dietary intervention1. Supplemental Table S4. Top 5 genera classified of microbiota recovered from BHI agar plates of pooled 4 kimchi samples purchased in 2021. Supplemental Table S5: Top 5 genera classified of microbiota recovered from BHI agar plates of pooled 4 cheese samples purchased in 2021. Supplemental Table S6: Top 5 genera classified of BHI-recovered microbiota of individual cheese samples purchased in 2022. Supplementary Table S7. Sensititre MIC for representative Gram-negative isolates from kimichi. Supplementary Table S8. Sensititre MIC for representative Gram-positive isolates from kimchi and artisan cheeses. Supplemental Table S9. Fecal antibiotic resistome of subjects by fermented foods intervention. Supplemental Table S10. Fecal antibiotic resistome of subjects by diets high in plant fibers.
