# Supplementary material for: High Prevalence of Antibiotic Resistance in Traditionally Fermented Foods as a Critical Risk Factor for Host Gut Antibiotic Resistome
Source: Microorganisms. 2024 Jul 15;12(7):1433. doi: 10.3390/microorganisms12071433 (PMC11279133; doi:10.3390/microorganisms12071433)
Supplement: Supplementary file 1 [file microorganisms-12-01433-s001.zip › microorganisms-3080559-supplementary.pdf]

Supplemental tables

Supplemental Table S1. Categories of foods consumed by subjects for dietary intervention.

	Food intervention (number of servings)							
Sub. ID	Cottage-Cheese	Kefir	Kombucha	Other-drinks	(Probiotic) Shots	Fermented Vegetables	Yoghurt	Total
8004		19.94	11.32	18.27		30.35	16.33	96.21
8008	1.85	1.08	2.67	8.89	6	10.66	4.45	35.6
8010			22.27			24.81	3.14	50.22
8011		2.66	6.65	6.53	7.62	21.92	8.42	53.8
8014		2.66	40.7				19.05	62.41
8016	1.23	3.17	17.35	15.21		48.58	20.74	106.28
8020			58.28				23.32	81.6
8021			16.02	9.34	0.58	53.44	17.5	96.88
8024		12	31.32			15.83	11.99	71.14
8025		6.73	12.91	5.16		15.49	12.43	52.72
8026		11.77	19.19			21.84	5.59	58.39
8027			41.9	5.06		15.78	13.33	76.07
8028		2.66	38.99		1.35	24.23	6.95	74.18
8030		26.64	22.43		24	84.71	21.93	179.71
8031	3	3.99	2.66		70	23.5	21.92	125.07
8032		11.97	22.24	6.33	33.36	1.52	5	80.42
8033			9.32			16.02	14.84	40.18
8034	3.39	15.31	17.35	16.4		7.6	11.47	71.52
Total	9.47	120.58	393.57	91.19	142.91	416.28	238.4	1412.4

Supplemental Table S2. Fermented food samples assessed in this study.

Sample #	Description	Year of Purchase	Plate recovered microbiota extraction shotgun	Direct sample extraction shotgun	AR isolate screening	AR isolate illustrated
Kimchi 1 (K1)	From Korean market A	2021	✓ (mixed)			
Kimchi 2 (K2)	From Korean restaurant 1	2021	✓ (mixed)			
Kimchi 3 (K3)	From Japanese restaurant 2	2021	✓ (mixed)			
Kimchi 4 (K4)	From Korean restaurant 3	2021	✓ (mixed)			
Kimchi 5 (K5)	From Korean restaurant 3	2022			✓	✓
Kimchi 6 (K6)	Bottled brand i, from national chain retail store B	2022			✓	
Kimchi 7 (K7)	From chain Korean restaurant 4	2022	✓	✓	✓	✓
Kimchi 8 (K8)	Bottled brand ii, from Asian market C	2022		✓	✓	✓
Kimchi 9 (K9)	Bottled brand iii, from Asian market C	2022		✓	✓	✓
Kimchi 10 (K10)	Bagged brand iv, from national chain retail store D	2022		✓	✓	✓
Kimchi 11 (K11)	From Japanese market E	2022		✓	✓	✓
Kimchi 12 (K12)	Bottled brand v, from Korean Market F	2022			✓	✓
Kimchi 13 (K13)	From Korean Market G	2022			✓	✓
Kimchi 14 (K14)	Bottled brand vi, from national chain retail store H	2023			✓	✓
Cheese 1 (C1)	Cambozola Blue, pasteurized milk (Germany)	2021	✓ (mixed)			
Cheese 2 (C2)	Comte, raw cow's milk (Aged > 60 days) (France)	2021	✓ (mixed)			

Cheese 3 (C3)	1924 Blue, pasteurized cow and sheep milk (France)	2021	✓ (mixed)			
Cheese 4 (C4)	Enrapture Rouge Blue, organic pasteurized milk (Unknown)	2021	✓ (mixed)			
Cheese 5 (C5)	Quadrello Di Bufala, pasteurized buffalo milk (Italy)	2022	✓		✓	✓
Cheese 6 (C6)	Pleasant Ridge Reserve, raw cow's milk (Aged > 60 days) (United States)	2022	✓		✓	✓
Cheese 7 (C7)	Thomasville Tomme, raw cow's milk (Aged > 60 days) (United States)	2022	✓		✓	✓
Cheese 8 (C8)	Swiss Raclette, pasteurized part-skim cow's milk (Switzerland)	2022	✓		✓	✓

Supplemental Table S3. Gut antibiotic resistome of subjects with dietary intervention[1].

	Total ARG copies/16S gene				
Subject ID	"Week-02"	Week0	Week08	Week10	Intervention by
8004	0.272186	0.284192	0.300493	0.239926	Fermented foods
8008	0.479228	0.508536	0.404436	0.527567	Fermented foods
8010	0.438165	0.460143	0.377093	0.408678	Fermented foods
8011	0.283978	0.241384		0.345206	Fermented foods
8014		0.367376	0.420609	0.411247	Fermented foods
8016	0.608693	0.498886	0.611231	0.591077	Fermented foods
8020	0.251731	0.287428		0.311607	Fermented foods
8021	0.357738	0.428821	3.77E-01	0.585925	Fermented foods
8024	0.345131	0.372126	0.364187	0.510634	Fermented foods
8025	0.188706	0.322113	0.308409	0.346233	Fermented foods
8026		0.33126	0.515311	0.446267	Fermented foods
8027	0.414694	0.332016	0.455848	0.347911	Fermented foods
8028	0.265774			0.351583	Fermented foods
8030	0.407724	0.514995	0.598983	0.448453	Fermented foods
8032	0.383879	0.416004	0.468486	0.718217	Fermented foods
8033	0.290082	0.272963	0.236222	0.23334	Fermented foods
8034	0.297562	0.364338	0.363455	0.287874	Fermented foods
8001		0.376419	0.316027	0.310167	High plant fibers
8002	0.274209	0.289648	0.264493	0.265522	High plant fibers
8003	0.624062	0.481308	0.447535	0.394988	High plant fibers
8006	0.219233	0.219967	0.233956	0.253023	High plant fibers
8009	0.182759	0.449417	0.415087	0.433871	High plant fibers
8017	0.765387	0.246743		0.342993	High plant fibers
8018	0.259782		0.241797	0.279882	High plant fibers
8022	0.364975		0.534551	0.44805	High plant fibers
8023		0.467738	0.385584	0.507301	High plant fibers
8029	0.397652	0.313326	0.326775	0.281955	High plant fibers
8036	0.381629	0.421726		0.45393	High plant fibers
8037	0.354639	0.320164	0.283361	0.384654	High plant fibers
8038	0.338513	0.269464	0.420975	0.311128	High plant fibers
8039	0.409468	0.552285	0.551152	0.555141	High plant fibers
8041	0.499627	0.501698	0.510558	0.526228	High plant fibers

[1]Numerical readings in total ARG copies per 16S gene.

Supplemental Table S4. Top 5 genera classified of microbiota recovered from BHI agar plates of pooled 4 kimchi samples purchased in 2021.

Sample	Top genera	Percentage
Mix_Kimchi_BHI	*Bacillus*	58.29%
	Serratia	13.42%
	Pseudomonas	12.11%
	Leuconostoc	9.92%
	Other	4.72%
	Lelliottia	1.55%
Mix_Kimchi_BHI_amp	*Pseudomonas*	46.63%
	Bacillus	41.71%
	Brucella	5.92%
	Enterobacter	2.71%
	Other	1.70%
	Rahnella	1.34%
Mix_Kimchi_BHI_tet	*Latilactobacillus*	34.33%
	Bacillus	33.42%
	Serratia	18.88%
	Stenotrophomonas	5.65%
	Lactococcus	4.14%
	Other	3.57%

Supplemental Table S5. Top 5 genera classified of microbiota recovered from BHI agar plates of pooled 4 cheese samples purchased in 2021.

Sample	Top genera	Percentage
Mix_Cheese_BHI	*Lactococcus*	47.32%
	Lacticaseibacillus	25.26%
	Staphylococcus	23.19%
	Other	2.04%
	Enterococcus	1.09%
	Streptococcus	1.09%
Mix_Cheese_BHI_tet	*Staphylococcus*	52.84%
	Bacillus	31.53%
	Streptococcus	12.24%
	Microbacterium	1.92%
	Other	1.29%
	Bacteroides	0.17%

Supplemental Table S6. Top 5 genera classified of BHI-recovered microbiota of individual cheese samples purchased in 2022.

Sample	Top genera	Percentage
Cheese5-BHI	*Mammaliicoccus*	64.41%
	Staphylococcus	18.62%
	Carnobacterium	5.26%
	Other	4.49%
	Glutamicibacter	3.73%
	Psychrobacter	3.51%
Cheese5-BHI-amp	Other	68.49%
	Stenotrophomonas	9.89%
	Pseudomonas	7.20%
	Burkholderia	5.91%
	Alcanivorax	5.31%
	Streptococcus	3.19%
Cheese5-BHI-tet	Other	64.56%
	Stenotrophomonas	11.03%
	Pseudomonas	7.46%
	Burkholderia	6.17%
	Alcanivorax	5.66%
	Streptococcus	5.14%
Cheese6-BHI	*Staphylococcus*	64.68%
	Enterococcus	22.47%
	Lacticaseibacillus	12.22%
	Other	0.29%
	Streptococcus	0.28%
	Leuconostoc	0.07%
Cheese7-BHI	*Staphylococcus*	73.00%
	Lactococcus	26.05%
	Leuconostoc	0.40%
	Other	0.32%
	Lacticaseibacillus	0.13%
	Streptococcus	0.11%
Cheese7-BHI-amp	Other	50.11%
	Pseudomonas	30.32%
	Burkholderia	5.63%
	Staphylococcus	5.18%
	Streptococcus	5.16%
	Leuconostoc	3.60%
Cheese7-BHI-tet	*Staphylococcus*	99.71%
	Other	0.10%
	Enterococcus	0.09%
	Leuconostoc	0.05%
	Mammaliicoccus	0.03%
	Lactococcus	0.02%
Cheese8-BHI	*Enterococcus*	44.89%

	Tetragenococcus	16.18%
	Staphylococcus	13.08%
	Lactococcus	12.68%
	Other	11.26%
	Carnobacterium	1.91%

Supplementary Table S7. Sensititre MIC for representative Gram-negative isolates from kimchi*.

Drug (Test Range, µg/mL)	*Kleb pneumoniae* K7-6	*Rahnella aquatilis* K11-3	*Serratia marcescens* K13-1
Amikacin (8-64)	**16**	<8	8
Ampicillin (4-32)	**>32**	**>32**	**>32**
Ampicillin/sulbactam 2:1 ratio (4/2-32/16)	8/4	<4/2	**>32/16**
Aztreonam (4-32)	<4	<4	**16**
Cefazolin (4-32)	4	**>32**	**>32**
Cefepime (4-32)	<4	<4	4
Cephalothin (2-16)	4	**>16**	**>16**
Meropenem (1-8)	<1	<1	**4**
Ertapenem (2-16)	<2	<2	**4**
Cefuroxime (4-32)	<4	**>32**	**>32**
Gentamicin (2-16)	4	<2	**>16**
Ciprofloxacin (0.5-4)	<0.5	<0.5	<0.5
Piperacillin/tazobactam constant 4 (16/4-128/4)	<16/4	<16/4	**>128/4**
Cefoxitin (4-32)	8	<4	**>32**
Trimethoprim/sulfamethoxazole (0.5/9.5-4/76)	<0.5/9.5	<0.5/9.5	**>4/76**
Cefpodoxime (2-16)	<2	**16**	8
Ceftazidime (1-32)	<1	<1	**>32**
Tobramycin (4-8)	<4	<4	>8
Tigecycline (1-8)	<1	<1	1
Ticarcillin/clavulanic constant 2 (16/2-64/2)	<16/2	<16/2	16/2
Ceftriaxone (1-64)	<1	**8**	**>64**
Tetracycline (0.5-16)	2	4	**>16**

*Bold MIC numbers: strongly indicative of the isolates being resistant to the corresponding antibiotics based on references (CLSI, 2020; Marchaim, 2014; Papich & Lindeman, 2018). *Italicized antibiotics:* penicillin and derivatives. *Italicized and highlighted antibiotics:* 4th generation of cephalosporin antibiotic (*Cefepime*) or Carbapenem antibiotics (*Meropenem*, *Ertapenem*).

Supplementary Table S8. Sensititre MIC for representative Gram-positive isolates from kimchi and artisan cheeses*.

Drug (Test Range, ug/mL)	*Lb. sakei* K5-1	*Lb. curvatus* K9-1	*Lb. sakei* K9-3	*Lb. brevis* K10-1	*Leuc. Citreum* K12-1	*Lb. brevis* K14-2	*Staphy xylosus* C6-2	*E. faecalis* C6-11
Erythromycin (0.25-4)	<0.25	>0.25	**>4**	<0.25	<0.25	<0.25	0.5	**>4**
Clindamycin (0.12-2)	<0.12	<0.12	>0.12	2	<0.12	<0.12	0.25	>2
Quinupristin/ dalfopristin (0.12-4)	>0.25	>0.25	>1	1	>0.5	0.5	0.5	>4
Daptomycin (0.25-8)	>4	>2	**>8**	**>8**	>0.25	**>8**	2	**>8**
Vancomycin (1-128)	**>128**	**>128**	**>128**	**>128**	**>128**	**>128**	1	**>128**
Tetracycline (2-16)	**>16**	**>16**	**2**	**16**	**>16**	**16**	**>16**	<2
Ampicillin (0.12-16)	>1	>1	>1	2	>0.5	2	0.25	1
Gentamicin (2-16/500)	**>16**	**>16**	**>16**	<2	>8	2	<2	**>16**
Levofloxacin (0.25-8)	**>8**	**>8**	**>8**	>2	>1	4	0.5	4
Linezolid (0.5-8)	>0.5	2	1	4	>2	4	2	4
Ceftriaxone (3rd) (8-64)	>8	>8	32	**>64**	<8	**>64**	8	**>64**
Streptomycin (1000)	<1000	**>1000**	**>1000**	<1000	<1000	<1000	<1000	<1000
Penicillin (0.06-8)	**>0.25**	**>0.25**	**0.5**	**8**	0.25	4	0.25	1
Rifampin (0.5-4)	2	<0.5	>1	0.5	>1	0.5	<0.5	4
Gatifloxacin (1-8)	**>4**	**>2**	**>8**	1	<1	1	<1	1
Ciprofloxacin (0.5-2)	**>2**	**>2**	**>2**	**>2**	**>2**	**>2**	0.5	**>2**
Trimethoprim/ sulfamethoxazole (0.5/9.5-4/76)	**>4/76**	**>4/76**	**>4/76**	**>4/76**	**>4/76**	**>4/76**	<0.5/9.5	**>4/76**
Oxacillin + 2%NaCl (0.25-8)	**>2**	**>4**	**>4**	**>8**	**>1**	**>8**	**1**	**>8**

*Bold MIC numbers: strongly indicative of the isolates being resistant to the corresponding antibiotics based on references (CLSI, 2020). *Italicized antibiotics:* penicillin and derivatives.

Supplemental Table S9. Fecal antibiotic resistome of subjects by fermented foods intervention.

ARG_types	8004_B2W	8004_0W	8004_8W	8004_10W	8008_B2W	8008_0W
aminoglycoside	0.049838	0.017378	0.086563	0.020008	0.038533	0.060416
bacitracin	0.012912	0.013694	0.015595	0.027826	0.025612	0.030658
beta-lactam	0.036763	0.016648	0.027417	0.016897	0.064398	0.040065
bleomycin	0	0	0	0	0	0
carbomycin	0	0	0	0	0	0
chloramphenicol	0.000317	0.003902	0.002014	0.001034	0.001264	0.001562
fosfomycin	9.92E-05	0	0	0	2.91E-05	0
fosmidomycin	0.002273	0.003419	0.000821	0.000453	0.000995	0.000402
fusaric-acid	0	0	0	0	0	0
fusidic-acid	0	0	0	0	0	0
kasugamycin	0	0	0	0	5.24E-05	0
macrolide-lincosamide-streptogramin	0.01064	0.027375	0.012997	0.008727	0.109551	0.149931
multidrug	0.005429	0.004625	0.006106	0.00522	0.005858	0.004065
polymyxin	0.000472	0	0.000152	2.83E-05	7.00E-05	0.000164
puromycin	0	0	0	0	0	0
quinolone	0.000331	0.001356	0.000486	0.000254	0	0
rifamycin	0	8.30E-05	0	0	3.29E-05	0
spectinomycin	0	0	0	0	0	0
sulfonamide	0.000109	0	0	1.23E-05	0.000346	0
tetracenomycin_C	0	0	0	0	0	0
tetracycline	0.122468	0.153039	0.099887	0.089917	0.209427	0.192851
trimethoprim	0	0	0	0	0	0
unclassified	0.000373	0.000402	0.000366	0.000373	0.002036	0.000817
vancomycin	0.030161	0.042271	0.048089	0.069177	0.021023	0.027604
SUM	0.272186	0.284192	0.300493	0.239926	0.479228	0.508536

8008_8W	8008_10W	8010_B2W	8010_0W	8010_8W	8010_10W	8011_B2W	8011_0W	8011_10W
0.040362	0.042148	0.006121	0.004569	0.007132	0.006964	0.008647	0.006303	0.010437
0.013811	0.01755	0.036304	0.040535	0.017888	0.02588	0.031929	0.034485	0.020079
0.068901	0.064998	0.087796	0.109024	0.087224	0.089338	0.066213	0.044235	0.088566
0	0	0	0	0	0	0	0	0
0	0	0	0	0	0	0	0	0
0.000253	0.001852	0.009375	0.008305	0.007467	0.008798	0.000462	0.003894	0.000153
0	0	0	0.000615	0	0	0	9.94E-05	0
0.000413	0.000916	0.000686	0.000595	0.000538	0.000627	0.002102	0.003026	0.002005
0	0	0	0	0	0	0	0	0
0	0	0	0	0	0	0	0	0
4.88E-05	0.000485	0.000396	0.00079	0.000103	0.000237	8.76E-05	0.000729	0.000103
0.08552	0.119767	0.062919	0.068587	0.06878	0.056612	0.017156	0.013175	0.022469
0.003398	0.010453	0.006997	0.008552	0.00425	0.00413	0.005641	0.01655	0.005736
0	0.000236	0.000222	0.000116	0	0	0.000134	0.000594	2.26E-05
0	0	0	0	0	0	0	0	0
0	0	0.000199	0.000105	0.000236	0.000111	0	7.01E-05	0
0	2.70E-05	0	0	0	4.66E-05	0	2.38E-05	0
0	0	0	0	0	0	0	0	0
0	0.00064	0	2.85E-05	0	0	0	0	0
0	0	0	0	0	0	0	0	0
0.179194	0.238526	0.214198	0.206891	0.175714	0.205469	0.140482	0.103002	0.178694
0	0	0	0	0	0	0	0	4.90E-05
0.000801	0.002606	0.002091	0.002568	0.000338	0.001194	0.000932	0.002969	0.00076
0.011734	0.027363	0.010859	0.008862	0.007424	0.009274	0.010193	0.01223	0.016133
0.404436	0.527567	0.438165	0.460143	0.377093	0.408678	0.283978	0.241384	0.345206

8016_B2W	8016_0W	8016_8W	8016_10W	8020_B2W	8020_0W	8020_10W	8021_B2W	8021_0W
0.022229	0.020013	0.034928	0.053946	0.0088	0.008774	0.010426	0.025025	0.02431
0.0215	0.024947	0.011508	0.018131	0.026811	0.030107	0.021996	0.018753	0.015698
0.005315	0.00599	0.030814	0.008353	0.039809	0.035977	0.042815	0.061037	0.083868
0	0	0	0	0	0	0	0	0
0	0	0	0	0	0	0	0	0
0.002805	0.004325	0.007127	0.004622	0.00034	0.001459	0.000822	7.16E-05	0.000216
0	0	0	0	0	0	0	0	5.73E-05
0.002285	0.001261	0.002698	0.001358	0.001116	0.003022	0.000354	0.001615	0.001389
0	0	0	0	0	0	0	0	0
0	0	0	0	0	0	0	0	0
0	0	0	0	0	0.001047	0.00013	7.75E-05	0.00011
0.062833	0.042229	0.073272	0.049231	0.007267	0.011724	0.008227	0.039288	0.054158
0.006647	0.005067	0.010103	0.00893	0.005118	0.027287	0.006476	0.005734	0.006474
2.25E-05	0.0001	1.76E-05	0.000102	0	0.000762	4.88E-05	5.34E-05	0.000201
0	0	0	0	0	0	0	0	0
0	0	0	0	0	0	0	0.000114	0.000112
0	0.000131	0	5.73E-05	0	0	0	0	0
0	0	0	0	0	0	0	0	0
0	0.000111	0.000128	0.000186	0	0	0	0.048849	0.045477
0	0	0	0	0	0	0	0	0
0.45042	0.366827	0.404997	0.408171	0.156819	0.154451	0.213953	0.141041	0.17557
0	0	7.56E-05	0	0	0	0	0	0
0.000448	0.000282	0.000979	0.001345	0.001396	0.007383	0.001435	0.000741	0.00117
0.034187	0.027604	0.034582	0.036644	0.004258	0.005436	0.004924	0.015338	0.02001
0.608693	0.498886	0.611231	0.591077	0.251731	0.287428	0.311607	0.357738	0.428821

8021_8W	8021_10W	8024_B2W	8024_0W	8024_8W	8024_10W	8025_B2W	8025_0W	8025_8W
0.021485	0.119437	0.030749	0.009134	0.017049	0.029256	0.013618	0.019032	0.017864
0.021255	0.018636	0.011112	0.0205	0.017149	0.014313	0.020335	0.013422	0.009863
0.036809	0.088092	0.051006	0.073257	0.066259	0.039388	0.00459	0.026535	0.02314
0	0	0	0	0	0	0	0	0
0	0	0	0	0	0	0	0	1.11E-05
0	0.000184	0.000169	0.000108	0.008072	0.014404	0.000262	0.000222	0
0	0	0.000152	1.85E-05	0	0	0	0	0
0.00162	0.000883	0.001456	0.001215	0.001681	0.004283	0.000435	0.001145	0.001618
0	0	0	0	0	0	0	0	0
0	0	0	0	0	0	0	0	0
8.50E-05	0.000141	0.000389	0.000602	0	0	0	0.000169	3.59E-05
0.058782	0.050691	0.046875	0.050701	0.06759	0.089968	0.005457	0.006331	0.007463
0.005931	0.005505	0.02187	0.016356	0.008626	0.006363	0.004824	0.010283	0.008341
0.000141	3.08E-05	0.000474	0.000548	0.000274	0.00055	4.19E-05	0.000617	0.000483
0	0	0	0	0	0	0	0	0
0.000122	2.16E-05	0.000201	0.000194	0	0	0	0	0
0	0	0	0	0	0	2.59E-05	0	0
0	0	0	0	0	0	0	0	0
0.011035	0.104362	0.000192	0.000509	0	0	0	0	4.56E-05
0	0	0	0	0	0	0	0	0
0.186868	0.181596	0.16549	0.179228	0.16759	0.295996	0.120352	0.226278	0.223252
0	0	0.000149	0.000376	0	0.000163	0	0	0
0.001159	0.001085	0.002372	0.003462	0.000585	0.000289	0.000249	0.000602	0.000716
0.031303	0.015261	0.012475	0.015917	0.009311	0.015662	0.018515	0.017478	0.015577
0.376594	0.585925	0.345131	0.372126	0.364187	0.510634	0.188706	0.322113	0.308409

8025_10W	8027_B2W	8027_0W	8027_8W	8027_10W	8028_B2W	8028_10W	8030_B2W	8030_0W
0.018384	0.011315	0.011032	0.007004	0.001977	0.006825	0.003313	0.003957	0.003492
0.011148	0.023201	0.029217	0.025933	0.039349	0.028681	0.019041	0.013653	0.018758
0.030871	0.082703	0.050974	0.082694	0.09616	0.022662	0.044527	0.041795	0.059637
0	0	0	0	0	0	0	0	0
0	0	0	0	0	0	0	0	0
0.000367	0.014278	0.011503	0.032713	0.007541	0.001417	0.001121	0.002984	0.002794
0	0	0.000112	7.68E-05	2.62E-05	0	0	0	0
0.001573	0.00287	0.000776	0.001422	0.000762	0.0012	0.001237	0.003467	0.00587
0	0	0	0	0	0	0	0	0
0	0	0	0	0	0	0	0	0
0.000159	0.000952	0.000564	0	0.000267	0.000297	0.00027	0.001849	0.003571
0.009449	0.043744	0.029784	0.062988	0.033243	0.023572	0.043125	0.007301	0.014773
0.015062	0.031113	0.020926	0.007413	0.013701	0.008387	0.003928	0.046509	0.099948
0.000617	0.00059	0.000601	0.000111	0.000225	9.65E-05	2.44E-05	0.00164	0.003446
0	0	0	0	0	0	0	0	0
0	0	0	0	0	0	0	0	0
0	0	0	0.000225	0	3.71E-05	2.29E-05	0	0
0	0	0	0	0	0	0	0	0
2.85E-05	0	0	0	0	0.000362	5.69E-05	0	0
0	0	0	0	0	0	0	0	0
0.240404	0.186418	0.152915	0.219362	0.142647	0.139937	0.218284	0.258655	0.269378
0	0	0	0	0	0.000178	2.11E-05	0	0
0.001586	0.004621	0.004196	0.00019	0.001852	0.001706	0.000833	0.011789	0.023284
0.016587	0.012887	0.019417	0.015714	0.01016	0.030414	0.015781	0.014125	0.010043
0.346233	0.414694	0.332016	0.455848	0.347911	0.265774	0.351583	0.407724	0.514995

8030_8W	8030_10W	8032_B2W	8032_0W	80326	8032_10W	8033_B2W	8033_0W	8033_8W
0.004206	0.005336	0.010154	0.012352	0.01859	0.020669	0.022823	0.038818	0.023776
0.021754	0.018914	0.030424	0.02973	0.020186	0.019895	0.021668	0.026406	0.019924
0.064547	0.04331	0.108333	0.104187	0.112423	0.194018	0.014785	0.007618	0.009444
0	0	0	0	0	0	0	0	0
0	0	0	0	0	0	0	0	0
0.004356	0.00638	0.000485	0.000443	0.0005	0.001759	0.001603	0.002098	0.000584
0	0	0	0	0	0	0	3.39E-05	7.96E-05
0.006871	0.003084	0.00255	0.003267	0.00387	0.001717	0.001637	0.000402	0.000615
0	0	0	0	0	0	0	0	0
0	0	0	0	0	0	0	0	0
0.005312	0.001134	1.43E-05	0.000317	0.000174	0.000237	0.001308	0.000203	0
0.011741	0.006792	0.037566	0.045211	0.080362	0.140909	0.019493	0.010098	0.00802
0.128124	0.030072	0.008182	0.0122	0.01051	0.007857	0.025762	0.008855	0.007294
0.004743	0.001156	0.000191	0.000384	0.000131	0.000292	0.000811	0.00036	0.000365
0	0	0	0	0	0	0	0	0
0	0	0	0	0	0	0	0	0
0	0	0	0	0	0	0	0	0
0	0	0	0	0	0	0	0	0
0	3.91E-05	0.000196	0.000548	0.000166	0.000414	0.001625	0.001696	0.001875
0	0	0	0	0	0	0	0	0
0.304266	0.307309	0.15508	0.183219	0.206756	0.312681	0.163946	0.150063	0.131815
0	0	0.00037	0.000435	0	0	0	0	0
0.031021	0.006699	0.001186	0.001832	0.001535	0.001196	0.00567	0.001559	0.001332
0.012044	0.018228	0.029148	0.021879	0.013282	0.016573	0.008951	0.024752	0.031098
0.598983	0.448453	0.383879	0.416004	0.468486	0.718217	0.290082	0.272963	0.236222

8033_10W	8034_B2W	8034_0W	8034_8W	8034_10W	8026_0W	8026_8W	8026_10W	8014_0W
0.032099	0.086401	0.114621	0.114143	0.074065	0.003999	0.00514	0.009192	0.013831
0.034706	0.018141	0.012035	0.01322	0.011101	0.023598	0.021598	0.016883	0.02
0.006796	0.00778	0.028169	0.017947	0.010226	0.075633	0.252866	0.183279	0.029829
0	0	0	0	0	0	0	0	0
0	0	0	0	0	0	0	0	0
0.000699	0.006226	0.003318	0.006683	0.006137	0.004355	0.005086	0.003395	0.001109
9.60E-05	0	0	0	0	0	0	0	0
0.000424	0.000335	0.000723	0.000358	0.000399	1.67E-05	5.25E-05	1.92E-05	0.001173
0	0	0	0	0	0	0	0	0
0	0	0	0	0	0	0	0	0
2.93E-05	0	0.000108	1.42E-05	0	0	0	1.71E-05	0
0.006336	0.04634	0.067716	0.067305	0.049594	0.048992	0.051172	0.017844	0.075317
0.00531	0.011028	0.007717	0.006096	0.011224	0.003433	0.004914	0.005123	0.004877
3.38E-05	0	8.97E-05	1.56E-05	0	2.21E-05	3.44E-05	7.06E-06	5.68E-05
0	0	0	0	0	0	0	0	0
0	0	0	0	0	0	0	0	0
0	0	0	0	0	0	0	2.43E-05	0
0	0	0	0	0	0	0	0	0
0.001199	0	0	0	0	0	0	0	0.000322
0	0	0	0	0	0	0	0	0
0.118135	0.103561	0.114939	0.125803	0.112519	0.144419	0.142764	0.169773	0.158462
0	0	0	0	0	0	0	0	0
0.000296	0.000425	0.000598	0.000275	0.00033	0.000325	0.000519	0.000302	0.000478
0.027179	0.017324	0.014304	0.011594	0.012279	0.026468	0.031164	0.04041	0.061922
0.23334	0.297562	0.364338	0.363455	0.287874	0.33126	0.515311	0.446267	0.367376

8014_8W	8014_10W
0.056465	0.026263
0.012715	0.030806
0.06502	0.028653
0	0
0	0
0.000895	0.000664
0	0
0.001562	0.001177
0	0
0	0
0	0.000339
0.077282	0.089888
0.005789	0.013432
2.48E-05	0.000496
0	0
1.51E-05	8.48E-06
1.51E-05	0
0	0
0.000662	4.01E-05
0	0
0.169782	0.154553
0	2.20E-05
0.000391	0.002167
0.02999	0.06274
0.420609	0.411247

Supplemental Table S10. Fecal antibiotic resistome of subjects by diets high in plant fibers.

ARG_types	8009_B2W	8009_0W	8009_8W	8009_10W	8041_B2W	8041_0W	8041_8W	8041_10W
aminoglyco	0.017951	0.045349	0.029031	0.03716	0.034937	0.043865	0.027263	0.033049
bacitracin	0.014153	0.024068	0.02261	0.040447	0.020147	0.017826	0.015201	0.016654
beta-lactan	0.012283	0.048176	0.054454	0.049828	0.078317	0.05107	0.083315	0.067393
bleomycin	0	0	0	0	0	0	0	0
carbomycir	0	0	0	0	0	0	0	0
chloramph	0.00138	0.002333	0.001527	0.001027	0.00156	0.000455	0	0.000512
fosfomycin	0	0	0	0	0.00E+00	0	0	0
fosmidomy	0.000932	0.002235	0.00195	0.001486	0.002678	0.002967	0.004026	0.002464
fusaric-acic	0	0	0	0	0	0	0	0
fusidic-acid	0	0	0	0	0	0	0	0
kasugamyc	0	0.000185	0.000355	2.30E-05	0.00E+00	0	4.65E-04	0
macrolide-l	0.027864	0.059653	0.069701	0.064788	0.130387	0.112107	0.132589	0.135412
multidrug	0.002959	0.004994	0.005897	0.004992	0.002662	0.00921	0.015924	0.003406
polymyxin	1.84E-05	2.90E-05	6.78E-05	9.49E-06	0.00E+00	0	0.000499	0
puromycin	0	0	0	0	0	0	0	0
quinolone	0	0	0	0	0	0.000143	0.004269	0
rifamycin	0	0	0	0	0.00E+00	7.51E-05	0	0.00E+00
spectinomy	0	0	0	0	0	0	0	0
sulfonamid	0.001146	0.00199	0.002458	0.001363	0.006115	0.003042	0.003977	0.013617
tetracenon	0	0	0	0	0	0	0	0
tetracycline	0.093841	0.240322	0.208632	0.211553	0.199122	0.22006	0.190779	0.23013
trimethopr	0	0	0	0	0	9.58E-05	0.001505	0
unclassified	0.000277	0.000557	0.00119	0.000315	0.000139	0.000724	0.003708	0.000353
vancomycir	0.009957	0.019526	0.017214	0.020881	0.023564	0.040059	0.027037	0.023238
SUM	0.182759	0.449417	0.415087	0.433871	0.499627	0.501698	0.510558	0.526228

8038_B2W	8038_0W	8038_8W	8038_10W	8017_B2W	8017_0W	8017_10W	8037_B2W	8037_0W
0.052453	0.016903	0.036582	0.036787	0.0167	0.007699	0.00971	0.052423	0.028569
0.011498	0.02088	0.022867	0.018901	0.042928	0.022556	0.029687	0.020654	0.021097
0.02146	0.009822	0.020975	0.019126	0.045067	0.049148	0.080903	0.040738	0.028462
0	0	0	0	0	0	0	0	0
0	0	0	0	0	0	0	0	0
0.001047	0.000461	0.000587	0.000595	0.006355	0.001437	0.002573	0.001039	0.001395
0	0	0	0	0	0	0	0	0
0.003717	0.002361	0.003405	0.002103	0.013636	0.000927	0.000852	0.00043	0.001539
0	0	0	0	0	0	0	0	0
0	0	0	0	0	0	0	0	0
7.39E-05	0	0.000308	0	0.01365	0.00031	7.17E-05	3.21E-05	0.000374
0.070872	0.040765	0.065668	0.052661	0.061069	0.022559	0.066639	0.053121	0.034061
0.003126	0.005005	0.006826	0.003223	0.33417	0.013823	0.006444	0.005906	0.011427
0.000165	0.000182	0.000147	0.000127	0.011983	0.000345	0.000341	1.25E-04	0.000194
0	0	0	0	0	0	0	0	0
0	0	0	0	0.001375	0.003071	6.61E-05	0	0
0	0	0	0.00E+00	0	0	0	0	0
0	0	0	0	0	0	0	0	0
0.000882	9.77E-04	0.000435	0.000692	0.014285	0.001376	0	0	0
0	0	0	0	0	0	0	0	0
0.163941	0.160417	0.24305	0.16526	0.100834	0.098916	0.125105	0.168911	0.177608
0	0	0	0	0.004021	0.000383	0	0	0
0.000157	0.000324	0.001441	0.000141	0.078715	0.002116	0.000656	0.000857	0.002463
0.009121	0.011369	0.018685	0.011512	0.0206	0.022079	0.019944	0.010403	0.012974
0.338513	0.269464	0.420975	0.311128	0.765387	0.246743	0.342993	0.354639	0.320164

8037_8W	8037_10W	8036_B2W	8036_0W	8036_10W	8029_B2W	8029_0W	8029_8W	8029_10W
0.038102	0.047054	0.011905	0.015572	0.003533	0.051956	0.030459	0.026638	0.024156
0.022058	0.021635	0.022238	0.014588	0.018714	0.032348	0.016111	0.024728	0.01843
0.026846	0.048199	0.034649	0.054258	0.069753	0.031508	0.034856	0.042315	0.030006
0	0	0	0	0	0	0	0	0
0	0	0	0	0	0	0	0	0
0.0007	0.000778	0.000182	0.000341	0	8.77E-03	0.002617	0.008467	0.011809
0	0	0	0.000251	0	0	0.00E+00	0	0
0.000662	0.001344	0.001228	0.001531	0.002604	0.002077	0.001672	0.001921	0.002029
0	0	0	0	0	0	0	0	0
0	0	0	0	0	0	0	0	0
0.000105	4.55E-05	4.42E-05	4.13E-05	0	3.95E-05	0.000104	1.45E-05	8.17E-05
0.038649	0.059769	0.056465	0.073207	0.078655	0.042873	0.026733	0.023625	0.022783
0.00605	0.006572	0.004791	0.004497	0.003512	0.009044	0.005051	0.004026	0.007611
0.00E+00	2.05E-05	8.13E-05	0.000101	0.00E+00	5.96E-06	2.52E-05	0	8.68E-05
0	0	0	0	0	0	0	0	0
0	0	0.000271	3.92E-05	1.95E-05	0	0	0	0.00E+00
0	0.00E+00	0	0	0	0	0	0	0
0	0	0	0	0	0	0	0	0
0	0	3.44E-05	7.2E-05	7.12E-05	3.9E-05	0	0	0
0	0	0	0	0	0	0	0	0
0.13829	0.189239	0.235827	0.241631	0.262887	0.201681	0.177969	0.177067	0.15381
0.00E+00	0	9.34E-05	0	0	3.3E-05	0	0	0
0.001421	0.001392	0.000661	0.001022	0.000286	0.000735	0.000892	0.00014	0.000471
0.010478	0.008607	0.013158	0.014574	0.013895	0.016546	0.016837	0.017835	0.010681
0.283361	0.384654	0.381629	0.421726	0.45393	0.397652	0.313326	0.326775	0.281955

8002_B2W	8002_0W	8002_8W	8002_10W	8003_B2W	8003_0W	8003_8W	8003_10W	8039_B2W
0.011026	0.00634	0.010514	0.007812	0.101759	0.055531	0.056162	0.03094	0.010795
0.012955	0.035643	0.01843	0.047663	0.013725	0.015179	0.012235	0.013799	0.029236
0.033407	0.033729	0.02913	0.023528	0.033142	0.073877	0.082018	0.140024	0.050688
0	0	0	0	0	0	0	0	0
0	0	0	0	0	0	0.00E+00	0	0
0.000387	0.00026	0.000142	0.000386	0.009381	0.00568	0.006642	0.00311	0.000157
0	0.00E+00	0	0	0.003238	0.001607	0.002193	0.001179	0
0.000825	0.00051	0.00101	0.001161	0.001249	0.000885	0.001384	0.001493	0.000485
0	0	0	0	0	0	0	0	0
0	0	0	0	0	0	0	0	0
0.000255	0	2.65E-05	0	0.000181	0	1.60E-04	0.000373	0.000109
0.028509	0.025388	0.02444	0.018549	0.073534	0.054946	0.044122	0.029885	0.07748
0.013215	0.004796	0.004722	0.004221	0.006072	0.004343	0.004129	0.003103	0.007786
0.000442	0	4.39E-05	0	1.67E-04	0.000169	0.000114	1.71E-05	7.27E-05
0	0	0	0	0	0	0	0	0
1.03E-05	0	0	0	0	0	0	0	0
0	0	0	0	0.00E+00	0	0	0	0
0	0	0	0	0	0	0	0	0
0.00071	0	0	0	0.000177	0.000765	6.06E-04	2.72E-04	0
0	0	0	0	0	0	0	0	0
0.160623	0.161081	0.163504	0.139511	0.323506	0.212959	0.186425	0.151616	0.218654
0	0	0	0	0	0	0	0	0
0.003177	0.000988	0.000622	0.000305	0.000462	0.000502	0.000654	0.001153	0.00088
0.008667	0.020912	0.011908	0.022386	0.057469	0.054865	0.050691	0.018024	0.013125
0.274209	0.289648	0.264493	0.265522	0.624062	0.481308	0.447535	0.394988	0.409468

8039_0W	8039_8W	8039_10W	8006_B2W	8006_0W	8006_8W	8006_10W	8001_0W	8001_8W
0.018165	0.008036	0.014238	0.011363	0.007024	0.019454	0.011582	0.021815	0.030157
0.024801	0.03393	0.02391	0.020021	0.01814	0.025746	0.025591	0.020398	0.025485
0.090773	0.107531	0.126718	0.013801	0.018815	0.004441	0.017358	0.076979	0.037186
0	0	0	0	0	0	0	0	0
0	0	0	0	0	0	0	0	0
0.0009	0.000251	0.000514	0.003994	0.002253	0.014463	0.004912	0.003115	0.001923
0	0.00E+00	0.00E+00	0	0	0	0	0	0
0.001385	0.001244	0.000545	0.002277	0.003118	0.001142	0.002683	7.66E-04	2.24E-03
0	0	0	0	0	0	0	0	0
0	0	0	0	0	0	0	0	0
0.000462	0.000232	0	7.75E-05	0	8.05E-05	0.000102	0	0
0.125935	0.125023	0.132023	0.004383	0.005591	0.00459	0.010953	0.044885	0.02781
0.022549	0.013	0.002176	0.003995	0.002877	0.004324	0.004463	0.008895	0.004608
0.000733	0.000277	0	6.44E-05	2.41E-05	4.81E-05	0.000225	2.78E-05	4.20E-05
0	0	0	0	0	0	0	0	0
0	0	0	9.93E-06	7.76E-05	0.000448	0.0001	0.000245	0.000308
0	0	0	0	0	0	0	0	0
0	0	0	0	0	0	0	0	0
0	0	0	0.000247	3.42E-05	0	9.06E-05	0	0.000178
0	0	0	0	0	0	0	0	0
0.251607	0.249684	0.247437	0.139534	0.147967	0.129128	0.152511	0.168752	0.152184
0	0	0	4.1E-05	0	4.78E-05	0	0	0
0.005159	0.002231	0.000238	0.000879	0.000276	0.000475	0.001027	0.000705	0.00065
0.009815	0.009715	0.007343	0.018546	0.013768	0.029569	0.021426	0.029834	0.033258
0.552285	0.551152	0.555141	0.219233	0.219967	0.233956	0.253023	0.376419	0.316027

8001_10W	8023_0W	8023_8W	8023_10W	8018_B2W	80186_8W	8018_10W	8022_B2W	8022_8W
0.026864	0.008388	0.018973	0.007669	0.005082	0.000795	0.000714	0.018066	0.053259
0.029305	0.01808	0.023963	0.021696	0.019425	0.018306	0.0186	0.009919	0.020146
0.030727	0.12621	0.061971	0.148234	0.032357	0.062896	0.084634	0.02637	0.029169
0	0	0	0	0	0	0	0	0
0	0	0	0	0	0	0	0	0
0.00198	0.000448	0.000366	5.27E-05	0.001032	0.002663	0.003006	0.000108	0.000583
0	0	0	0	0	0	0	0	0
1.06E-03	0.001389	0.001057	0.001378	0.000727	0.000482	0.000631	0.002393	0.005965
0	0	0	0	0	0	0	0	0
0	0	0	0	0	0	0	0	0
1.60E-04	0.000182	0.000203	0.000516	0.000232	3.48E-05	3.50E-05	0.001523	0.004161
0.040247	0.021998	0.053068	0.022141	0.023743	0.022435	0.016271	0.029797	0.037001
0.005629	0.007336	0.004115	0.00332	0.010579	0.002537	0.003363	0.0341	0.117701
3.07E-05	2.32E-04	0.00E+00	9.77E-05	0.000378	0	2.26E-05	0.001304	0.003624
0	0	0	0	0	0	0	0	0
0.00021	0	0.00E+00	0.00E+00	3.71E-05	0.000308	0.00062	0	0
0.00E+00	0	0.00E+00	0	0	0	0	0	0
0	0	0	0	0	0	0	0	0
0.000411	0.000458	1.74E-05	4.36E-05	0.000404	0.000154	7.36E-05	0.00329	0.007895
0	0	0	0	0	0	0	0	0
0.137074	0.267121	0.194696	0.285508	0.148497	0.12318	0.142695	0.211351	0.199801
0	0.000223	0	7.50E-05	0	0	0	0.001317	0.006573
0.000768	0.001515	0.000646	0.001441	0.00232	0.00065	0.000886	0.009418	0.026291
0.035701	0.014157	0.02651	0.015128	0.014968	0.007357	0.008329	0.016018	0.022383
0.310167	0.467738	0.385584	0.507301	0.259782	0.241797	0.279882	0.364975	0.534551

8022_10W
0.039421
0.014617
0.031086
0
0
0.000316
0
0.00343
0
0
0.003272
0.040499
0.071975
0.002506
0
0
0
0
0.00533
0
0.195577
0.003831
0.017703
0.018487
0.44805
